# Cognitive behavioral therapy with adaptive virtual reality exposure vs. cognitive behavioral therapy with *in vivo* exposure in the treatment of social anxiety disorder: A study protocol for a randomized controlled trial

**DOI:** 10.3389/fpsyt.2022.991755

**Published:** 2022-10-10

**Authors:** Per Trads Ørskov, Mia Beck Lichtenstein, Mathias Torp Ernst, Iben Fasterholdt, Asge Frederik Matthiesen, Marco Scirea, Stephane Bouchard, Tonny Elmose Andersen

**Affiliations:** ^1^Research Unit for Digital Psychiatry, Mental Health Services in the Region of Southern Denmark, Odense, Denmark; ^2^Department of Clinical Research, University of Southern Denmark, Odense, Denmark; ^3^Centre for Innovative Medical Technology, Odense University Hospital, Odense, Denmark; ^4^Maersk Mc-Kinney Moller Institute, University of Southern Denmark, Odense, Denmark; ^5^Department of Psychoeducation and Psychology, University du Québec en Outaouais, Gatineau, QC, Canada; ^6^Department of Psychology, University of Southern Denmark, Odense, Denmark

**Keywords:** social anxiety, cognitive behavioral therapy, virtual reality, exposure, psychophysiological measurements, heart rate, electrodermal activity, machine learning

## Abstract

**Background:**

Social anxiety disorder (SAD) has a high prevalence and an early onset with recovery taking decades to occur. Current evidence supports the efficacy of cognitive behavioral therapy (CBT) with virtual reality (VR) exposure. However, the evidence is based on a sparse number of studies with predominantly small sample sizes. There is a need for more trials investigating the optimal way of applying VR based exposure for SAD. In this trial, we will test the efficacy of CBT with adaptive VR exposure allowing adjustment of the exposure based on real-time monitoring of the participants's anxiety level.

**Methods:**

The trial is a randomized controlled, assessor-blinded, parallel-group superiority trail. The study has two arms: (1) CBT including exposure *in vivo* (CBT-Exp), (2) CBT including exposure therapy using individually tailored VR-content and a system to track anxiety levels (CBT-ExpVR). Treatment is individual, manual-based and consists of 10 weekly sessions with a duration of 60 min. The study includes 90 participants diagnosed with SAD. Assessments are carried out pre-treatment, mid-treatment and at follow-up (6 and 12 months). The primary outcome is the mean score on the Social Interaction Anxiety Scale (SIAS) with the primary endpoint being post-treatment.

**Discussion:**

The study adds to the existing knowledge by assessing the efficacy of CBT with adaptive VR exposure. The study has high methodological rigor using a randomized controlled trial with a large sample size that includes follow-up data and validated measures for social anxiety outcomes.

**Clinical trial registration:**

ClinicalTrials.gov, identifier: NCT05302518.

## Introduction

Social anxiety disorder (SAD) is a common anxiety disorder characterized by excessive fear of being scrutinized or criticized by others leading to avoidance of social situations (1). According to the ICD-10 classification of mental and behavioral disorders, engaging in feared situations is accompanied by autonomic symptoms of anxiety such as sweating, trembling or increased heartrate (HR). The lifetime prevalence of SAD ranges between 8.4 and 12.1% and the 12 month prevalence ranges between 4.2 and 7.1% (2, 3).

The avoidance of social interaction affects both work and personal life (4). Several social situations may be avoided because of excessive fear, such as small talk with peers or relatives, attending a job interview, expressing opinions or giving a presentation at work (4). Social anxiety may occur as a consequence of having high standards for one's own performance in social situations or having a wish to present oneself in a desired way combined with a lack of confidence in achieving these standards or making the right impression (5).

SAD is related to reduced health-related quality of life (6) and is also associated with substantial psychiatric comorbidity including other anxiety disorders, mood disorders and substance use disorders (3, 7). Epidemiological studies show that SAD most often precedes depression and that SAD is related to a substantial and consistent increase in risk of subsequent depression (8). Similarly, symptoms of social anxiety often precedes alcohol dependence (7).

SAD is an adolescent-onset disorder with a long recovery period (3, 8). Despite the prolonged recovery, few individuals with SAD seek treatment for their disorder. Only about one-third of lifetime cases report ever seeking treatment for SAD (3). Not seeking treatment may be related to the nature of the disorder itself. Individuals with SAD avoid treatment because the treatment itself constitutes a social situation that provokes anxiety (9).

### Treatment

The treatment of choice for social anxiety is cognitive behavioral therapy (CBT) (4). Treatment is conducted both individually and in group-settings. Exposure therapy is central to CBT and is very effective in fear reduction (10). Different theoretical approaches to cognitive behavioral therapy and exposure therapy exist; including inhibitory learning and emotional processing theory (11, 12). The inhibitory learning paradigm emphasizes exposure therapy which provides a foundation for learning alternative contingency rules competing with the conditioned fear response related to social situations (11). Emotional processing theory stresses the importance of habituation happening as a results of exposure as the driving mechanism leading to improvements in treatment (12).

A prominent cognitive behavioral model for SAD is the one proposed by Clark and Wells (13). According to their model, SAD is maintained by cognitive processes in addition to certain strategies and behaviors applied by the individual with SAD, including safety behaviors, inner focus of attention, anticipatory and post-event processing, and dysfunctional assumptions. Therapy focuses on addressing these maintaining factors and therefore therapy makes extensive use of behavioral experiments including in session role-play and *in vivo* exposure during sessions or conducted as homework assignments between sessions.

*In vivo* exposure is effective when treating SAD (14), but conducting *in vivo* exposure in session can be challenging because relevant social situations might be difficult to obtain and control. In addition, finding the right setting for exposure can be time consuming and costly (15, 16).

### Virtual reality and exposure-based therapy

Virtual reality computer-generated environments or 360° videos presented in a head mounted display that interacts with head movement creates the illusion of being able to look and move around in a virtual world. In 360° videos, the participant is immersed in a spherical video with 3 degrees of freedom (DoF). The interaction with head movement in 360° videos is constrained to rotation about the three perpendicular axes (pitch, yaw and roll) (17). Whereas, computer-generated environments may have 6 DoF making also translational movement possible in VR (i.e., moving forward/backward, up/down, left/right) (17). Hand controllers may further add to the immersive qualities of rendered environments by enabling interaction with the virtual environment.

VR has been used for exposure across different anxiety disorders and generally, there is no evidence that VR exposure is less efficacious than exposure *in vivo* (18–20). Exposure in virtual reality has several advantages compared to *in vivo* exposure. Virtual reality provides readily available environments for exposure, such as a meeting room with a group of people waiting for the patient to give a presentation. Furthermore, exposure in VR is highly controllable and can be modified to fit the needs of the patient. Finally, exposure takes place confidentially within the safety of the therapy room and thus the threshold for initiating exposure might be lower than for *in vivo* exposure.

A handful of randomized controlled trials (RCT) have implemented virtual reality exposure as part of the treatment for SAD (21–24) and for public speaking anxiety (25). Overall, the studies show that CBT with exposure in VR has a superior effect compared to waitlist control, and similar effect when compared to CBT with *in vivo* exposure. However, one study by Bouchard et al. was able to show, that exposure in VR was superior to *in vivo* exposure when embedded in CBT leading them to conclude, that VR-exposure embedded in CBT is an efficient and cost-effective way to treat SAD (22). Generally, the RCTs suffer from small sample sizes, with one larger study having 97 participants, but most studies being smaller having between 69 and 45 participants. There is a need for more RCTs investigating the effect of VR-exposure, especially larger RCT's.

Several meta-analyses on VR-exposure for SAD have been conducted (19, 26–30). Post-treatment the effect of CBT with VR-exposure is significant when compared to waitlist control, and the effect size is around g = 0.80. Whereas, the difference between CBT with VR-exposure and CBT with *in vivo* exposure is insignificant with effect sized ranging between g = 0.07 and g = −0.27.

### Psychophysiological measures in an adaptive treatment

Adaptive virtual scenarios may show additional clinical benefits by giving the therapist increased control in regulating the intensity of the exposure according to current anxiety levels (31–33). However, the effect of adaptive virtual scenarios applied in therapy remains largely unexplored (31). Real time assessment of anxiety level based on physiological measures may provide an essential aid in the therapist decisions on the progression of the exposure treatment (32). Detecting anxiety using psychophysiological measures has the advantage that it enables frequent assessment without disturbing the participants' sense of presence in VR (the illusion of being *there*) (34). Moreover, psychophysiological measures reflect involuntary reactions of the body, and are difficult to mask (35).

Psychophysiological measures such as electrodermal activity (EDA) and electrocardiogram (ECG) or photoplethysmograph (PPG) can be used to estimate anxiety in real time during exposure (32). EDA measures the changes in skin conductance that changes due to eccrine sweat production. Eccrine sweating reflects the sympathetic activity of the autonomic nervous system (ANS) and happens in the response to emotional stimuli like stress, anxiety, fear or pain (36). ECG or PPG can be used to measure HR. Parasympathetic and sympathetic activity of the ANS both affect the HR. Increased levels of sympathetic activity results in higher levels of HR. Like eccrine sweating, HR are used in emotion detection (35).

Using data derived from measures of EDA and PPG Petrescu et al. were able to predict low, medium and high levels of anxiety in real-time during exposure for heights (32). They developed a regression model utilizing different variables derived from EDA and PPG. Assessing anxiety levels using Subjective Units of Distress Scale (SUDS) they obtained a high accuracy between estimated anxiety, and actual anxiety levels (e.g., SUDS score), with an accuracy ranging between 69.52 and 90.48%.

There is a need for more trials investigating the optimal way of applying VR-exposure for SAD. The study described in the present protocol adds to the existing knowledge by assessing the efficacy of adaptive VR-exposure. Estimating anxiety levels based on psychophysiological measures provides the therapist the opportunity to increase or decrease the intensity of the exposure ensuring that intensity of the exposure is optimal. Thus, adaptive VR-exposure may provide additional clinical benefits compared to regular VR-exposure. The study is having high methodological rigor by being a RCT with a large sample size that includes follow-up data and well-validated measures for social anxiety outcomes and follows the SPIRIT guidelines for high quality RCTs (37).

### Objectives and hypotheses

In participants diagnosed with SAD, we aim to compare the effect of CBT including exposure therapy using individually tailored VR-content and a system to track anxiety levels (CBT-ExpVR) to CBT including exposure *in vivo* (CBT-Exp). Both treatments will be conducted as individual therapy.

#### Primary hypothesis

At post treatment we expect that CBT including exposure therapy using individually tailored VR-content and a system to track anxiety levels (CBT-ExpVR) will result in lower levels of social anxiety than CBT with exposure *in vivo* (CBT-Exp). The outcome on social anxiety will be measured using the total score on Social Interaction Anxiety Scale (SIAS).

#### Secondary hypotheses

- At 6 and 12 months follow-up, we expect that VR-treatment will result in lower levels of social anxiety than *in vivo*-treatment.
- Post treatment and at 6 and 12 months follow up we expect that VR-treatment will result in lower levels of depression and higher levels of self-rated health than *in vivo*-treatment.
- The dropout rate we expect will be lower for the VR-treatment compared to the *in vivo*-treatment.

In addition to the evaluation of effect, a health economic evaluation will be made from a societal perspective.

## Methods

This protocol is written in accordance with SPIRIT 2013 (37, 38) and the TIDieR checklist and guide (39).

### Design

The trial is a randomized controlled, assessor-blinded, parallel-group superiority trail. The study is conducted at the Center for Digital Psychiatry in the Mental Health Services in the Region of Southern Denmark. Participants are randomly assigned to two different conditions: 1) CBT-ExpVR and 2) CBT-Exp. Both conditions offer a 10-week program with weekly one-hour sessions.

### Participants and recruitment

Participants will be recruited from the whole country but primarily from the Region of Southern Denmark. Different strategies will be used for recruitment including online advertisements (Facebook, Instagram, Twitter, LinkedIn, the Center for Digital Psychiatry's homepage, Sundhed.dk, and the project's own website), newspaper ads in addition to flyers and/or posters distributed at upper secondary schools, higher education, drop-in centers, and clinics of general practitioners.

Participants are referred to the trial's website where they are provided with written information about the study and are invited to complete online questionnaires screening for social anxiety symptoms and symptoms of depression. The questionnaire consists of SIAS and Major Depression Inventory and questions on current treatment and use of medication. Cut-off score for inclusion is <22 on SIAS (40, 41) and cut-off score for exclusion on MDI is <29. The online questionnaire might be supplemented by phone calls to inquire further information on current treatment and medication. No information will be obtained from patient records.

**Figure 1:**
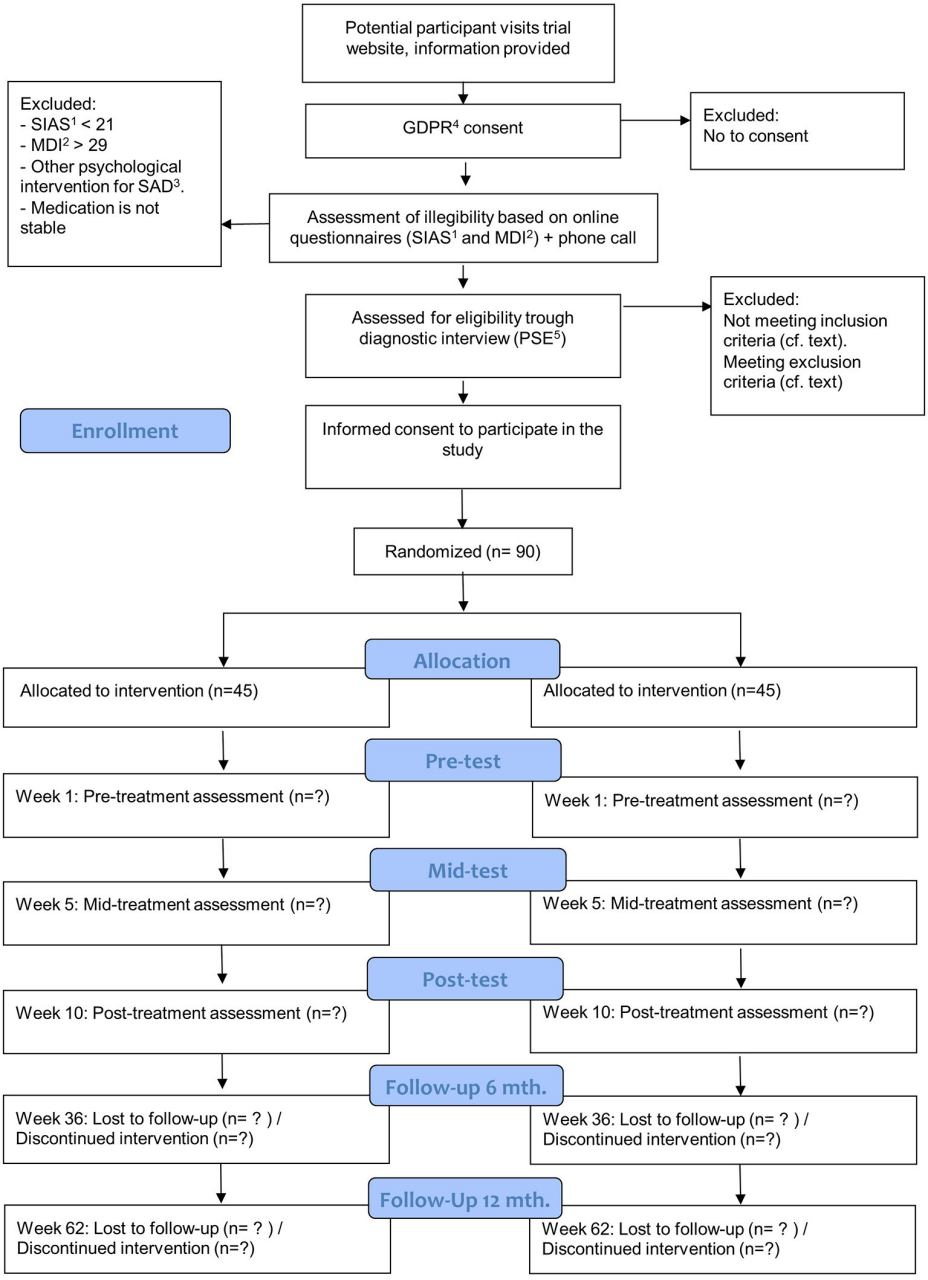
Flowchart for inclusions, interventions, and assessments. ^1^SIAS, Social Interaction Anxiety Scale; ^2^MDI, Major Depression Inventory; ^3^SAD, Social Anxiety Disorder; ^4^GDPR, General Data Protection Regulation; ^5^PSE, Present State Examination.

Eligible participants are invited to an assessment at the Center for Digital Psychiatry. Before the assessment, participants will receive thorough verbal information about the participation in the trial from one of the psychologists responsible for the treatment. At the assessment the participant will have the opportunity to bring a person of his or her own choice. The assessment will be carried out using the short version of the Present State Examination (PSE). At the assessment participants will be questioned on their use of medication, especially if they use any prescribed medicine that may affect HR or eccrine sweat production. Difficult cases will be discussed with a supervisor and/or at a weekly clinical conference before the diagnosis is made. Participants who meet the inclusion criteria will be offered to participate in the study. Participants who do not meet the inclusion criteria for the study and need treatment are recommended to contact their general practitioner as well as other available resources. Before the pre-treatment assessment, included participants will be asked to complete an informed consent form. With information about the trail provided at the diagnostic interview participant will have more than the 24 h to consider whether to enroll in the study or not. Figure 1 provides an overview of the recruitment process, interventions, and assessment.

### Inclusion criteria

Participants must meet the following inclusion criteria:

- Age 18–75.
- Sufficient knowledge of the Danish Language.
- Fulfilling the diagnostic criteria for SAD according to ICD-10 classification of mental and behavioral disorders (F 40.1) (1).

### Exclusion criteria

- Previously diagnosed with autism spectrum disorders.
- Previously diagnosed with psychotic disorders.
- Severe depression (>29 MDI).
- Dependence syndrome according to ICD-10 classification of mental and behavioral disorders (F1x.2) (1).
- Suicidal ideation.
- Dementia/Intellectual disability.
- Epilepsy.
- Taking part in other kinds of psychological intervention for SAD.
- Medication (SSRI, benzodiazepine, MAOI) type and doses needs to be stable three months prior to inclusions and during the intervention.

### Randomization and blinding

Participants are block randomized (1:1) using random block size. The block sizes will not be disclosed, to ensure concealment. Computer generated random numbers using the platform Sealed Envelope (https://www.sealedenvelope.com) will be used to generate the allocation sequence. The allocation sequence is handled by a data manager from the Patient data Explorative Network (OPEN) and is unavailable to those who enroll and assign participants. We will use the randomization module in REDCap (Research Electronic Data Capture) to assign participants. To prevent foreknowledge of treatment assignment to affect enrollment, the psychologist responsible for the diagnostic interview is blind to treatment assignment. Participants are blinded to their treatment assignment until their first treatment session. Participants will only be blind to treatment assignment at the pre-test but not at later assessments. A clinician blind to treatment assignment will administer the assessment taking place at pre-test, mid-test, and post-test.

### Sample size

A recent study reported ~10-point drop in SIAS for standard treatment and 20-point drop for traditional VR-exposure with standard deviations around 15 points (22). With adaptive/tailored VR-exposure we find it reasonable to assume a similar effect size (d = 0.66 and 1.33, respectively). The standard variation is between participants, which we expect to be conservative for our paired design. Using the measurement error based on test-retest reliability of the SIAS a drop of 13 points is considered a statistical reliable change (41). Meta-analyses have indicated smaller effect sizes, but the analyses are still based on rather few RCTs, which did not benefit from the improvements we propose in this study. If 35 participants are recruited for each group, this will lead to a statistical power of 0.80 comparing the VR-treatment to in vivo-treatment at the 0.05 significance level. To consider a 20% dropout we plan to invite 90 participants in total.

### Interventions

Interventions are carried out by psychologists with at least one full year of clinical experience. Before the study therapists are trained delivering treatment in accordance with the treatment manual. The manual is reviewed with the principal investigator and the different sessions of the intervention is practiced through role-play. Therapy sessions will take place at the Center for Digital Psychiatry with one therapy room for VR-treatment and one for *In vivo*-treatment.

#### VR-treatment

The intervention is an individual manual-based cognitive behavioral therapy with adaptive exposure in VR. The intervention consists of a 10-week treatment program with 1 h weekly session adapted from Clark and Wells (13, 42). The treatment rationale is based on a model of the maintenance of social phobia developed by Clark and Wells, and the treatment aimed at reversing the maintaining processes identified by the model (13). Sessions dedicated to exposure are scheduled from the fourth to the ninth session. The three first sessions are dedicated to building the therapeutic relationship and providing psychoeducation in addition to providing a rationale for exposure therapy. The themes of therapy are: (a) The general ideas of CBT, (b) The maintaining processes of social phobia, (c) negative automatic thoughts, (d) shifting focus of attention form self-focus to external focus, (e) safety behaviors, (f) post-processing, (g) self-processing, (h) schemas and rules for living. The last session is dedicated to evaluation and relapse prevention. In session and for homework assignments worksheets are used extensively to practice and reinforce the skills learned in therapy. The intervention utilizes homework assignments including *in vivo* exposure.

##### Exposure in VR

We will use 360° videos for exposure using an HTC VIVE Pro headset. The HTC VIVE Pro has a field of view of 110°, the resolution per eye is 1,440 × 1,600 pixels with a refresh rate of 90 Hz. It has 6 DoF tracking capabilities, however 360° videos only allow for experiencing 3 DoF. The 360° videos we will use for exposure was recorded using Insta360-InstaOneX and edited using the game engine Unity 3D.

Six different VR-scenarios will be used for exposure: (1) Taking a seat on a bench in a public park, (2) Being introduced as a new employee, (3) Performing a presentation at a meeting, (4) Entering and shopping in a grocery store, (5) Visiting a café, and (6) Using public transportation. VR exposure for SAD has so far predominately been conducted using computer-generated virtual environments (21–23). However, at least three studies conducted used 360° videos in the treatment of SAD (43–45). A feasibility study from our lab showed that 360° videos are able to trigger anxiety in individuals with SAD (46).

The scenarios were chosen in order to reflect different situational domains of social anxiety including informal speech/interaction, formal speech/interaction, observation by others and assertion (47). All scenarios consist of multiple scenes with both participant and therapist choices. The therapist can choose the length of the exposure as well as the difficulty of the exposure.

Participants will be seated during all exposures in VR and will interact with the environment through integrated eye-gaze options, most commonly utilized for movement (e.g., sitting down, entering a room) as well as indicating choices (e.g., “ask the shop assistant” or “await the shop assistant”). Other interaction options include dialogue, where the clinician controls scene changes and reactions from the environment, prompting a second question from the boss when the participant, as a new employee, is finishing answering the first one.

While the participant is in the VR-scenario, data on HR and EDA are collected using iMotions software. These data are collected to continuously estimate the anxiety experienced by the participant. The therapist can watch the VR-scenario on a monitor, while the participants are in VR. On the monitor, the participant's anxiety level will be displayed (low, medium and high), and act as a guide to aid the therapist decisions on how to conduct the exposure (i.e., the length and the intensity of the exposure). Continuous estimates of the participants anxiety level allows for frequent assessment without disturbing the participants' sense of presence in VR. Data on the participant's anxiety levels during exposure along with recorded video is available after the exposure and can be included as a tool in the therapy session.

The exposure treatment is personalized to each participant. The therapist and participant can choose which scenarios they find most useful in achieving treatment goals. It is allowed to use the same VR-scenarios for two or more exposure sessions.

#### In vivo-treatment

The intervention is an individual manual-based cognitive behavioral therapy. The intervention consists of a 10-week treatment program with 1 weekly session adapted from Clark and Wells (13, 42). Sessions dedicated to exposure *in vivo* are scheduled from the fourth to the ninth session. Exposure will take place at the Center for Digital Psychiatry and the surrounding areas. The therapist will plan the exposure with the participant and will accompany the participant during the exposure. Themes of the therapy are similar to the VR-treatment. *In vivo* exposure is also conducted as homework. The amount and intensity of the exposure during session and assigned as homework are matched between the two interventions. *In vivo* exposure is personalized based on treatment goals. The level of intensity of the exposure is based on the participants anxiety hierarchy.

#### Criteria for discontinuing allocated intervention

If participants fail to meet for an appointment, they will receive a letter to remind them of the agreed treatment plan. Participants, who fail to meet for appointments for treatment more than three times in total will be excluded from treatment.

#### Treatment fidelity

The interventions are manualized to increase treatment fidelity. To ensure that the treatment is delivered consistently and reliably in accordance with the manual the therapist will after each treatment session answer a self-report questionnaire on specific treatment targets for each session (48).

### Data collection and management

**Figure 2:**
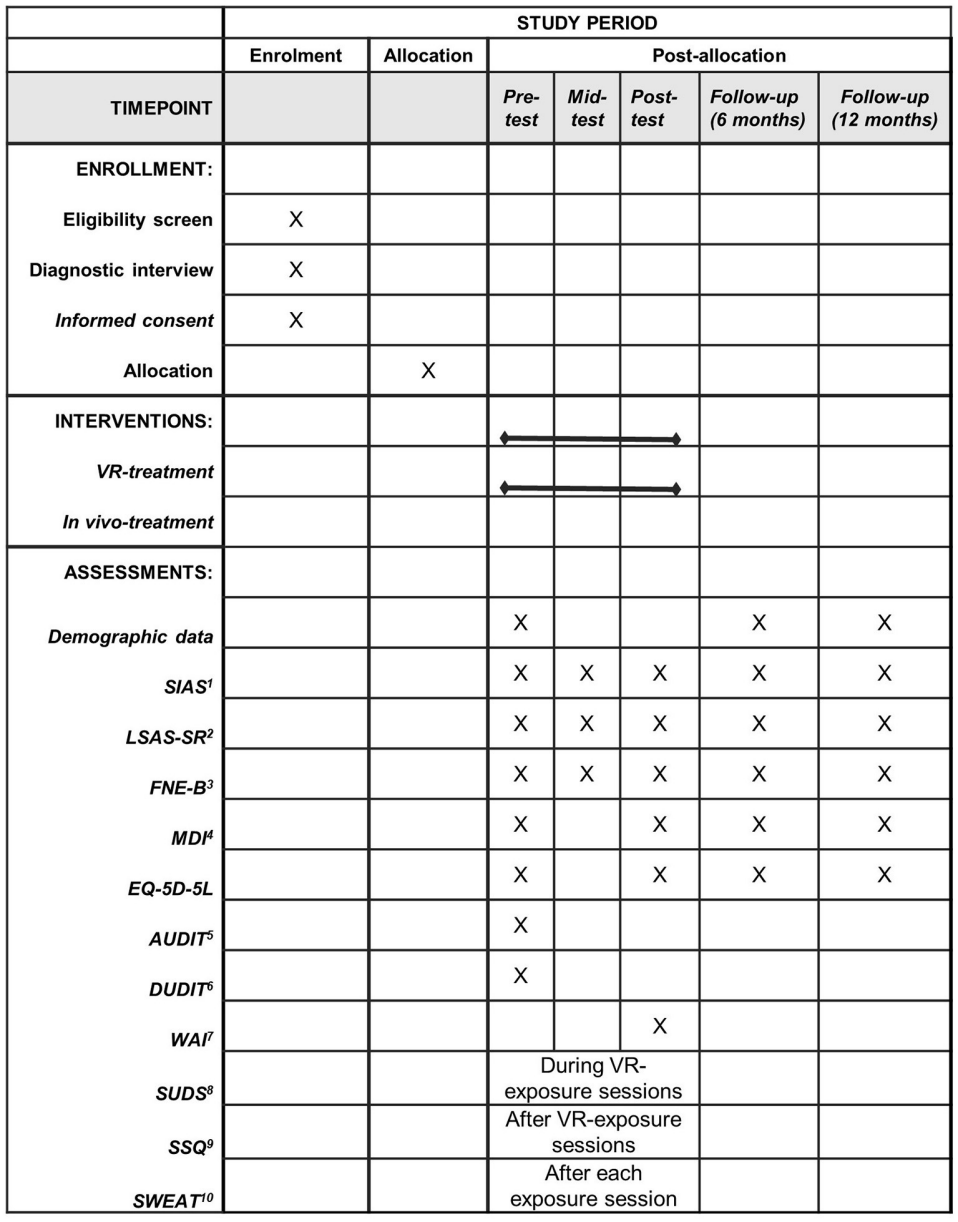
Schedule of enrollment, interventions, and assessments. ^1^SIAS, Social Interaction Anxiety Scale; ^2^ LSAS-SR, Leibowitz Anxiety Scale-Self report version; ^3^FNE-B, Fear of Negative Evaluation-Brief version; ^4^MDI, Major Depression Inventory; ^5^AUDIT, Alcohol Use Disorders Identification Test; ^6^DUDIT, Drug Use Disorders Identification Test; ^7^WAI, Working Alliance Inventory; ^8^SUDS, Subjective Units of Distress Scale; ^9^SSQ, Simulator Sickness Questionnaire; ^10^SWEAT, Specific Work for Exposure Applied in Therapy.

Data in relation to the primary and secondary outcomes will be collected at pre-treatment (week 1), mid-treatment (week 5), post-treatment (week 10) and at 6 months follow-up (week 36) and 12 months follow-up (week 62). Figure 2 provides an overview of the planned assessments. Data collected from the participants using self-report measures and data reported by therapist are collected using REDCap. REDCap is an electronic data capture tool and is hosted by OPEN, which is part of the REDCap Consortium. To ensure confidentiality assigned researchers and the data manager at OPEN will be the only people with access to data at REDCap. Informed consent forms will be scanned and stored in REDCap. Data is stored at OPEN's server located in the Regions of Southern Denmark. SUDS, HR, EDA, and estimated anxiety is collected using iMotions, and data is saved on a secure folder on SharePoint. Both REDCap and SharePoint complies with the Danish Data Protection Act and the General Data Protection Regulation (GDPR). Data from REDCap will be exported to statistical software (SPSS, Stata, and R) on OPEN's server located in the Region of Southern Denmark. Personal identifiers in the dataset have been flagged and will not be exported from REDCap to ensure confidentiality.

### Availability of data and materials

PTØ will have access to the full trial dataset. Data will be stored on OPEN's server located in the Region of Southern Denmark. February 2025 data will be transferred to The Danish National Archives. Data are available upon reasonable request. Restrictions apply to the availability of data and approval is needed from Danish Data Protection Agency and or The Danish National Archives.

### Assessment

#### Primary outcome measure

SIAS measured at pre-treatment, mid-treatment, post-treatment and at follow-up (6 and 12 months). The instrument is a measure of social anxiety symptoms and consists of 20 items assessing cognitive, affective, and behavioral responses to social interaction. In a comparison of social phobia outcome measures Cox et al. found strong support for SIAS which showed good sensitivity to treatment effects (49). The scale ranges from 0 to 80. Participants have to indicate to what degree they feel each statement is characteristic to them using a 5-point Likert scale (0 = not at all, 1 = slightly, 2 = moderately, 3 = very, 4 = extremely). The instrument has high internal consistency, Cronbach's alpha = 0.93, and high test-retest reliability, r = 0.92 (50).

#### Secondary outcome measures

Leibowitz Anxiety Scale-Self-report version (LSAS-SR) measured at pre-treatment, mid-treatment, post- treatment and at follow-up. LSAS-SR is a measure of anxiety and avoidance in a range of social situations (51).

Fear of Negative Evaluation-Brief version (FNE-B) measured at pre-treatment, mid-treatment, post-treatment and at follow-up. FNE-B is a measure of distress experiences when getting negative evaluations by others (52).

Major Depression inventory (MDI) measured at pre-treatment, post-treatment and at follow-up. MDI is a measure of symptoms severity in depression (53).

EQ-5D-5L measured at pre-treatment, mid-treatment, post-treatment and at follow-up. EQ-5D-5L is a measure of health status (54).

#### Other measures

Alcohol Use Disorders Identification Test (AUDIT) measured at pre-test. AUDIT is a screening for unhealthy alcohol use (55).

Drug use disorders identification test (DUDIT) measured at pre-test DUDIT is a screening instrument for drug-related problems (56).

Specific Work for Exposure Applied in Therapy (SWEAT) measured after each exposure session (session 3 to 9). SWEAT is answered by the therapist and measures costs and efforts required to conduct exposure (57).

Working Alliance Inventory (WAI) measured post-treatment. WAI measures the collaborative engagement of therapist and participant (58).

Subjective Units of Distress Scale (SUDS) will be measured during exposure in virtual reality at session 3 to 9. SUDS is used to measure intensity of anxiety experienced by the participant during exposure (59).

Heart rate (HR) is collected using an optical heart rate sensor (Polar Verity Sense). The heart rate sensor provides the heart rate in 1-s interval based on a sampling rate of 135 Hz. Data is collected using the iMotions software. HR will be collected during exposure in VR.

Simulator Sickness Questionnaire (SSQ) measured after each exposure session in VR. SSQ is answered by the participant after each exposure session in VR and it is a measure of simulator sickness (60).

Electrodermal Activity (EDA) is collected using BioPAC systems. Data is collected at a sampling rate of 1,000 Hz. Data is collected using the iMotions software. EDA will be collected during exposure in VR.

Estimated Anxiety is collected using iMotions software. The estimation of anxiety is based on HR and EDA measurements utilizing artificial neural networks (NeuroEvolution of Augmenting Topologies). Estimated anxiety (low 0–33 on SUDS, medium 34–66 SUDS, high 66–100 on SUDS) will be collected during exposure in VR.

### Statistical analyses

The primary statistical analysis will be carried out as intention to treat (ITT). We will use linear mixed models to analyze the data. Separate analysis will be performed for each outcome variable. A two-level model with observations nested within participants is used. The fixed effects will be time, intervention, and the interaction between time and intervention as well as the baseline score. In addition to the primary analysis, a per-protocol analysis will be carried out on those participants completing at least 50% of the exposure sessions, interpreting the results cautiously due to the potential selection bias and confounding. Linear mixed models produce accurate estimates under the assumption that data are missing at random (MAR). A sensitivity analysis will be carried out where missing data will be handled by multiple imputation (m=100). Imputations will be based on baseline characteristics and secondary outcomes used chained equations. The moderating effect of the working alliance, depressive symptoms, alcohol, and drug use on the treatment outcome will be explored as subgroup analysis with continuous moderators by including them as covariates interacting with treatment and time. Model validation in the linear mixed model will be performed by inspection of qq-plots of residuals and best linear unbiased predictors to assess normality, and plotting residuals against fitted values to check homoscedasticity. If assumptions are violated, analysis will be performed after log-transformation. If assumptions do not hold under log-scale, bootstrapping will be applied with the linear mixed model, taking bootstrap sampling clustered by participant, and estimating post-treatment effect using bias-corrected accelerated confidence intervals (61).

### Health economic evaluation

Health economic evaluation will be made from a societal perspective. The types of resources included in the analysis will be based on a review by Kidholm and Kristensen (62) and include:

Program costs:

○ Fixed costs: Hardware and software, training of the psychologists,
○ Variable costs: Number of consultations and duration of consultations.

Economic consequences:

○ Sick leave (absence from study or work)
○ Changes in labor market attachment
○ Contacts to GP, psychologist, hospital, emergency ward
○ Participants time when doing homework assignments.

Except for the fixed costs, data will be collected from the participant and from health registers (the Danish National Patient Register, National Health Insurance Service Register). Reporting of the study will follow the CHEERS guideline by Husereau, Drummond (63).

### Monitoring

Since the trial has a short duration and presents a minimal risks to the participants, no interim analysis is planned, and no data monitoring committee will be assigned. Cybersickness similar to motion sickness may occur in the VR-setting. The VR-exposure scenarios are designed in a way to minimize cybersickness, and the exposure sessions are brief which also diminishes the risk of experiencing cybersickness. Cybersickness will be monitored using the Simulator Sickness Questionnaire. Adverse events will be registered by the therapists after each therapy session.

### Ancillary and post trail care

Participants that are enrolled into the study are covered by indemnity for negligent harm through The Patient Compensation Association (Patienterstatningen). There may be a burden associated with answering questionnaires. In order to increase motivation and acknowledge efforts to answer questionnaires for research use, the participants are rewarded with a gift card worth 500 DKK after treatment. This is only rewarded when the participant completes the treatment and is taxable.

### Dissemination policy

Results will be disseminated regardless of the magnitude or direction of effect. Both positive, negative, and inconclusive results will be made public and both beneficial and harmful effects of adaptive virtual reality exposure therapy will be reported. Dissemination will happen in scientific journals, at scientific conferences, as well as via www.clinicaltrials.gov. Authorship will be determined according to the Vancouver Guidelines. Recruitment will start in March 2022. Treatment will be carried out during 2022 and until the spring of 2023. Follow-up will be finished spring 2024. A report on trial results will be submitted in 2023. In addition, the results will be made public via the press, the webpage of the Center of Digital Psychiatry and social media.

## Discussion

The study described in this protocol will assess the efficacy of adaptive VR-exposure applied in a CBT framework. The effect of adaptive virtual scenarios utilizing psychophysiological measures to estimate anxiety levels in therapy remains unexplored (31). The study is a randomized controlled trial with a large sample size that includes follow-up data and validated measures for social anxiety outcomes. The present study will add to the existing knowledge on VR-exposure used in therapy. The project will be of great importance if it confirms that the treatment is suitable for individuals with SAD. Very few individuals with SAD end up ever seeking treatment. For this individual with SAD, VR-reality interventions might lower the threshold for seeking treatment. We need different treatment options and more effective treatments, which is particularly important, because SAD starts out at a young age with recovery taking decades to occur.

Concerning practical and operational issues involved in performing the study, we know from previous research that recruiting participants for SAD can be challenging. Based on our experience we have developed a deliberate plan for recruitment. Halfway through the recruitment period (June 2022), we will evaluate our progress and make adjustments to the recruitment procedures if needed.

## Ethics statement

This study has been approved by the Medical Research Ethics Committees in Denmark, *de Videnskabsetiske Medicinske Komiteer* (Case number: 2170848). Important protocol modifications will be communicated to the Medical Research Ethics Committees. Participants receive oral and written information before they are asked to give their written informed consent to participate in the study.

## Author contributions

ML conceived the original idea for the study and wrote the application for the Innovation Fund Denmark. PØ developed the study design and wrote the draft for the protocol. ME and AM developed the six VR-scenarios. MS developed the model for estimating the anxiety levels during exposure in VR. IF is responsible for the health economic evaluation following the trail. TA, IF, SB, and ME contributed to the development of the design. All authors contributed to refinement of the study protocol and approved the final manuscript.

## Funding

The VR8 research program was initiated by ML, Head of Research, Center for Digital Psychiatry, Mental Health Services in the Region of Southern Denmark. ML submitted an application to the Innovation Fund Denmark and VR8 was granted 12.4 million DKK. ML has no affiliation to Innovation fund Denmark. The VR8 research program consists of different work packages including software development, development of VR-videos for exposure, general product development, VR8 - feasibility study, VR8 – randomized controlled trial (present study), implementation, and finally a cost-effectiveness analysis. The Center for Digital Psychiatry, University of Southern Denmark, Odense University Hospital, and iMotions A/S are partners in the VR8 research program and support the program through co-financing. Co-financing amounts to 3.9 million DKK and covers part of the salaries for researchers and other personnel working on the project. Innovation Fund Denmark will not have any role in the design of the study, execution, analysis and interpretation of the data or in the writing of manuscripts and the decision to submit results.

## Conflict of interest

SB is the president and owns equity in Cliniques et Dévelopment in Virtuo, which develops virtual environments, and conflicts of interest are managed according to Université du Québec en Outaouais conflict of interest policy. Cliniques et Dévelopment in Virtuo did not create the virtual environments use in this study. The remaining authors declare that the research was conducted in the absence of any commercial or financial relationships that could be construed as a potential conflict of interest.

## Publisher's note

All claims expressed in this article are solely those of the authors and do not necessarily represent those of their affiliated organizations, or those of the publisher, the editors and the reviewers. Any product that may be evaluated in this article, or claim that may be made by its manufacturer, is not guaranteed or endorsed by the publisher.
